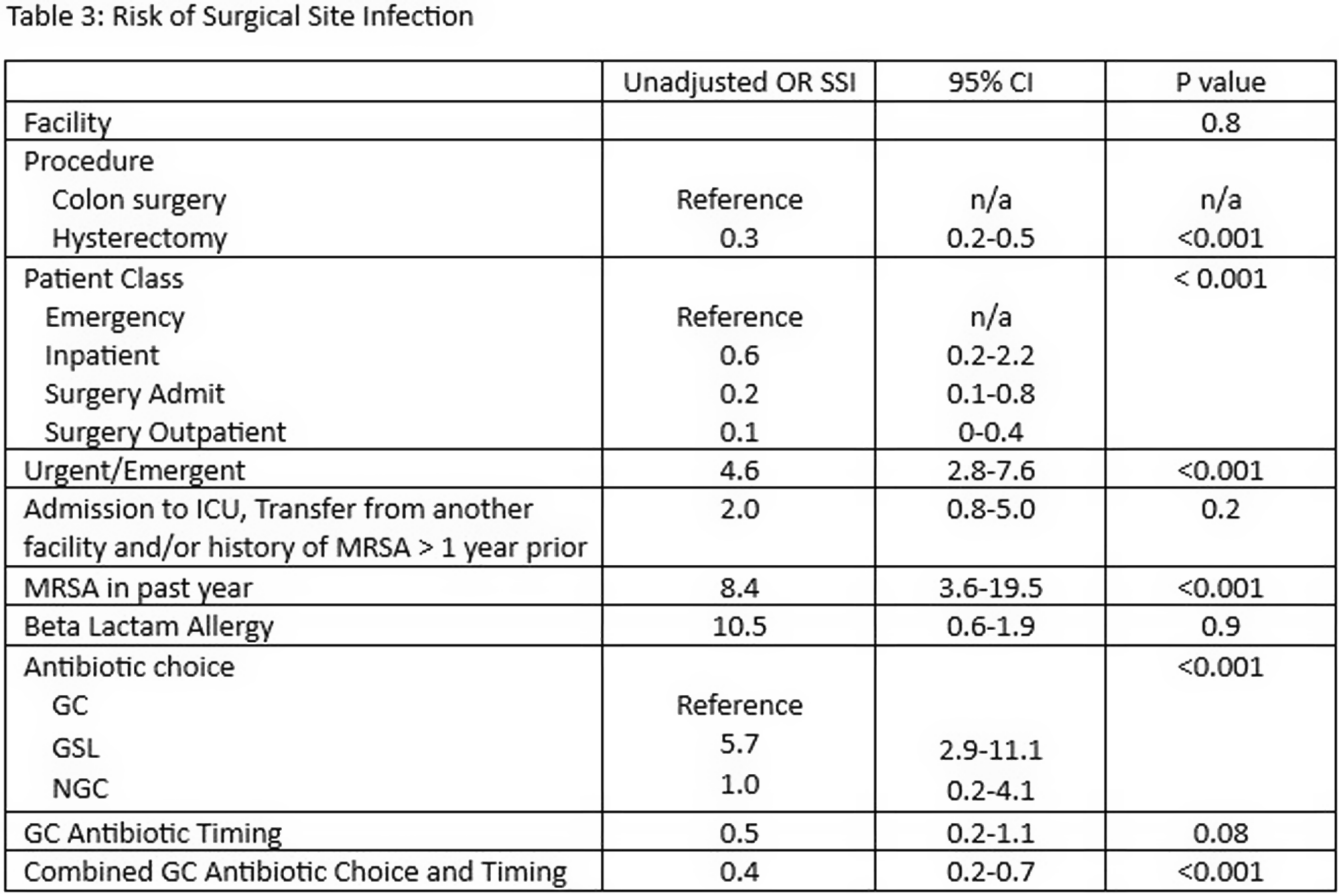# Concordance with Preoperative Intravenous Antibiotics Guidelines and Risk of Surgical Site Infection (SSI)

**DOI:** 10.1017/ash.2024.319

**Published:** 2024-09-16

**Authors:** Anupama Neelakanta, Lisa Davidson, Kristin Fischer, Shelley Kester, Jennifer Onsrud, Katie Passaretti

**Affiliations:** Carolinas HealthCare System; Atrium Health

## Abstract

**Background:** Administration of antimicrobial prophylaxis close to incision time is recommended as an essential practice to prevent surgical site infections (SSI). Despite guideline recommendations, adherence to preoperative intravenous antibiotic guidelines is variable. We aim to assess perioperative factors associated with guideline concordant (GC), guideline second line (GSL) and non-guideline concordant (NGC) antibiotic choice and timing and impact on odds of SSI. **Methods:** 3173 patients at 9 hospitals with National Health Safety Network (NHSN) procedure codes for colon surgery and abdominal hysterectomy between January 1, 2023, and October 31, 2023, were identified from the electronic medical record. Data on preoperative intravenous antibiotic choice and timing, history of allergy and methicillin resistant staphylococcus aureus (MRSA) history was collated. SSI were identified using NHSN definitions by trained infection preventionists. SSI identified as present at the time of surgery were excluded. Antibiotic choice and timing were compared to institutional guidelines and patients were categorized as having received GC, GSL or NGC antibiotic choice, and GC or NGC antibiotic timing. Descriptive statistics were used to describe clinical data for patient population and subgroups who received GC, GSL and NGC antibiotics. Univariate logistic regression was performed to assess the association of procedural and clinical factors with the likelihood of an SSI. **Results:** GC was high overall for both antibiotic choice (94%) and timing (94%). (Table 1) NGC and GSL antibiotic choice were more common in colon surgery, urgent/emergent cases, patients with beta lactam allergies and those with a recent history of MRSA. GSL antibiotic choice was more frequent in inpatients. (Table 2). GC antibiotic timing was more common with GC antibiotic choice (98%) than with NGC (37%) or GSL (51$) antibiotic choice. Odds of SSI were lower in patients who were GC for both antibiotic choice and timing (OR 0.4, p < 0 .001) and increased in patients who received GSL antibiotic choice (OR 5.7 compared to GC, p < 0 .001), underwent urgent/emergent surgeries (OR 4.6, p < 0 .001) or had a history of MRSA in the past year (OR 8.4, p < 0 .001). We found a non-significant trend toward lower infections in patients with GC antibiotic timing (OR 0.5, p 0.08). (Table 3) **Conclusion:** Combined GC for antibiotic choice and timing was high and associated with lower odds of SSI. NGC and GSL antibiotic choice were associated with patient level factors such as history of MRSA and emergent procedures which may also impact risk of SSI.

**Figure:**
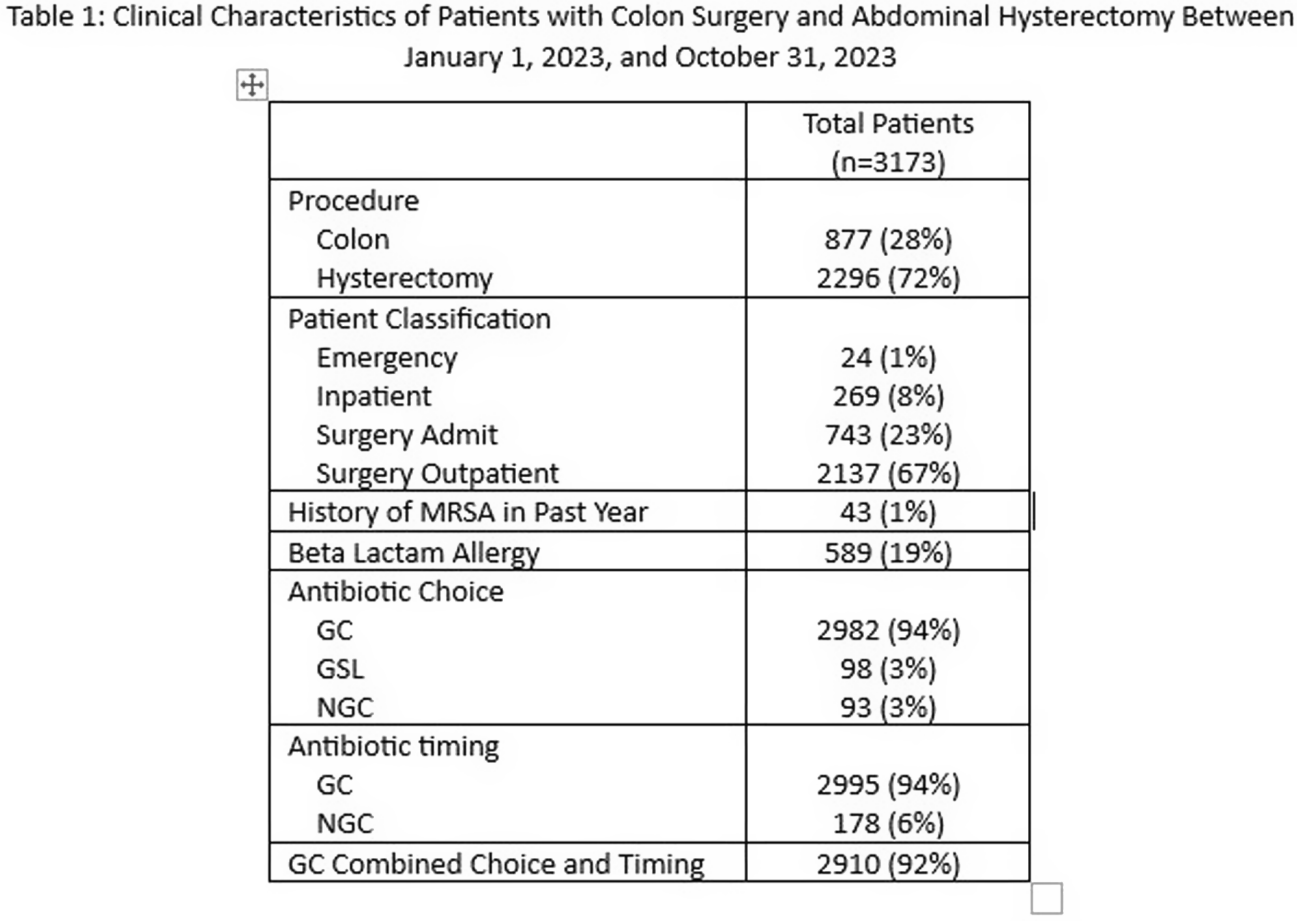

**Figure:**
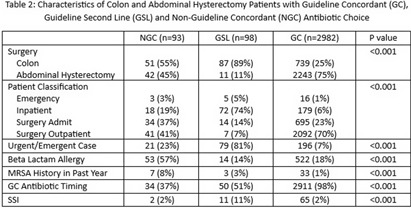

**Figure:**